# Inositol Requiring Enzyme (IRE), a multiplayer in sensing endoplasmic reticulum stress

**DOI:** 10.1080/19768354.2021.2020901

**Published:** 2022-01-10

**Authors:** Zhixin Zhou, Qian Wang, Marek Michalak

**Affiliations:** Department of Biochemistry, University of Alberta, Edmonton, Canada

**Keywords:** IRE1α, unfolded protein response, endoplasmic reticulum, stress sensor

## Abstract

The endoplasmic reticulum (ER) can sense a wide variety of external and internal perturbations and responds by mounting stress coping responses, such as the unfolded protein response (UPR). The UPR is composed of three stress sensors, namely IRE1α, PERK, and ATF6 that are activated to re-establish ER homeostasis. IRE1α represents the most ancient branch of the UPR affecting many cellular processes in plant and animal cells. IRE1α is a type I transmembrane protein with kinase/nuclease activities in response to ER stress. Both the ER luminal and cytosolic IRE1α interactomes have been identified revealing a multifunctional role of the ER stress sensor. IRE1α is also associated with organellar membrane contacts to promote rapid communication between intracellular organelles under stress conditions.

## Introduction

Responses to stress are an integral part of an organism’s physiology and biology. To deal with stress cells have evolved various mechanisms; the success or failure of these mechanisms depends to a large extent on the nature and duration of the stress. The endoplasmic reticulum (ER) is a large, dynamic em and one of the largest components of the cellular reticular network (CRN) (Michalak and Agellon 2018; Wang et al. 2019). The ER plays many vital roles in the cell including Ca^2+^ storage, protein synthesis, folding and post-translational modification, phospholipid and steroid synthesis, and stress responses (Schroder and Kaufman 2005; Schroder 2008; Lam and Galione 2013; Schwarz and Blower 2016; Wang and Kaufman 2016). The ER continuously communicates with other components of the CRN including the Golgi apparatus, nucleus, and mitochondria; mediates lipid synthesis, Ca^2+^ and inflammatory signaling, and transcriptional regulation (Phillips and Voeltz 2016; Lombardi and Elrod 2017). Not surprisingly, disruption of ER function caused by intrinsic and extrinsic factors culminates in ER stress, with the ER initiating a coping response [e.g. unfolded protein response (UPR)], to mitigate the stress (Groenendyk, Sreenivasaiah, Kim, et al. 2010; Walter and Ron 2011; Kraskiewicz and FitzGerald 2012; Chen and Brandizzi 2013; Groenendyk et al. 2013; Grootjans et al. 2016; Wang and Kaufman 2016; Hetz and Papa 2018; Gonzalez-Quiroz et al. 2020; Hetz et al. 2020; Urra et al. 2020). The ER, therefore, is an important component of CRN that allows cells to adjust to a wide variety of conditions. The UPR pathway can sense disturbance in protein folding in the ER and involves distinct components designed to re-establish the protein synthetic machinery, including translational attenuation, transcriptional activation of genes encoding chaperones and components of the ER-associated degradation (ERAD), and activation of apoptotic and autophagy pathways (Kraskiewicz and FitzGerald 2012; Groenendyk et al. 2013; Grootjans et al. 2016; Gonzalez-Quiroz et al. 2020; Urra et al. 2020; Wang and Kaufman 2016; Hetz and Papa 2018).

There are three integral ER membrane proteins, stress sensors, and signal transducers: the ER kinase dsRNA-activated protein kinase-like ER kinase (PERK), activating transcription factor 6 (ATF6), and inositol-requiring enzyme 1 (IRE1) that in combination with the ER molecular chaperone immunoglobulin binding protein (BiP), they comprise the UPR response to ER stress (Groenendyk et al. 2013; Hetz and Papa 2018). BiP interacts with IRE1α, PERK, and ATF6 but upon stress, BiP is sequestered away from the stress sensors, allowing activation of the UPR pathways (Demay et al. 2014; Yukimoto et al. 2021; Groenendyk, Sreenivasaiah, Kim, et al. 2010; Walter and Ron 2011; Chen and Brandizzi 2013; Groenendyk et al. 2013; Grootjans et al. 2016; Gonzalez-Quiroz et al. 2020; Hetz et al. 2020; Urra et al. 2020).

Many excellent reviews have been published on the UPR signaling (Groenendyk, Sreenivasaiah, Kim, et al. 2010; Walter and Ron 2011; Chen and Brandizzi 2013; Groenendyk et al. 2013; Grootjans et al. 2016; Gonzalez-Quiroz et al. 2020; Hetz et al. 2020; Urra et al. 2020). IRE1α the most ancient branch of the UPR affects many cellular processes in plant and animal cells (Groenendyk, Sreenivasaiah, Kim, et al. 2010; Groenendyk et al. 2013). Here we focus on selected aspects of the IRE1α structure, function, and regulation. Recent work also places IRE1α signaling as an important factor in physiology and pathology of the cardiovascular system (Groenendyk, Sreenivasaiah, Kim do, et al. 2010; Groenendyk et al. 2013; Glembotski 2014; Groenendyk et al. 2016; Arrieta et al. 2017; Groenendyk et al. 2020).

## IRE1, the gene, and the protein

**Figure 1.**
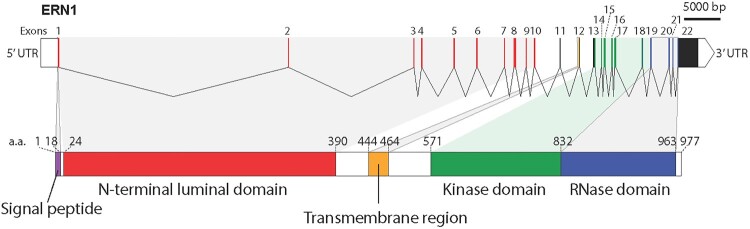
The IRE1α gene and protein. Human IRE1α encoded by the ERN1 gene, consists of 22 exons and 93,390 bases. The IRE1α protein consists of signal peptide, N-terminal luminal domain (NLD), a signal helix transmembrane domain, and cytoplasmic region containing kinase and RNase activity.

The IRE1 gene was originally identified by complementation of a yeast mutant auxotrophic for inositol and subsequently characterized as a serine/threonine protein kinase required for myo-inositol synthesis (Nikawa and Yamashita 1992). Since then, IRE1α has been identified as a component of the UPR signaling pathway important for sensing and responding to ER stress in a variety of eukaryotic organisms (Chen and Brandizzi 2013; Grootjans et al. 2016; Gonzalez-Quiroz et al. 2020; Urra et al. 2020; Li and Howell 2021; Siwecka et al. 2021). In mammals, there are two homologs of IRE1, IRE1α, and IRE1β encoded by two genes, *ERN1* and *ERN2*, respectively (Figure 1). IRE1α is expressed in all cells, whereas IRE1β is expressed predominantly in the intestinal epithelium (Zhou et al. 2006). IRE1β is restrictively expressed in the gut and IRE1β knockout mice are viable (Tirasophon et al. 2000; Tsuru et al. 2013).

Interestingly, whole-body IRE1α deficiency in mice is embryonic lethal at E9.5-11.5 in mice due to placental malformation (Iwawaki et al. 2009). However, whole-body gene knockout of the *Xbp1* gene, which encodes the transcription factor induced by the ‘canonical’ activation of IRE1α signaling, is embryonic lethal at E12.5-14.5 due to impaired hepatocyte development and hepatic hypoplasia (Reimold et al. 2000). The observed delay in the onset of lethality exhibited by whole-body XBP1-deficient mice relative to the whole-body IRE1α-deficient mice supports the notion that IRE1α is involved in regulating functions in addition to those associated with XBP1 splicing.

**Figure 2.**
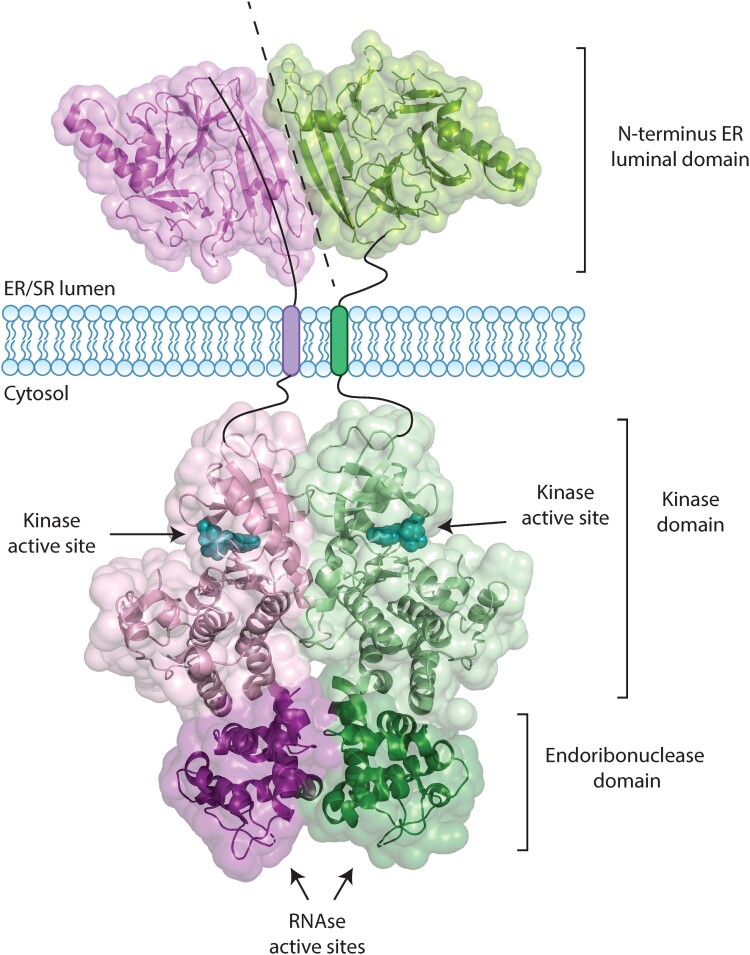
Structure of the IRE1α dimer. IRE1α is a type I transmembrane protein that consists of an N-terminal domain facing the ER/SR lumen, a single transmembrane domain, and a cytosolic domain with kinase and endoribonuclease activity. The figure shows two monomers of IRE1α, in purple and green, with solvent-accessible surfaces. The luminal domain of IRE1α (PDB: 2HZ6) forms a stable dimer by hydrogen bonding and hydrophobic interactions; the dimer interface is marked by the dashed line. The cytosolic domain of IRE1α (PDB: 2RIO) contains a kinase domain and endoribonuclease (RNase) shown in a back-to-back arrangement, which is suggested to be the RNase active states. The arrows indicate the location of the kinase and RNase active site. ADP molecules bound to the kinase active sites are shown in cyan.

Both IRE1 homologs are type I transmembrane proteins with kinase/nuclease activities triggered by oligomerization of IRE1α in response to ER stress (Tirasophon et al. 2000; Li et al. 2010). IRE1α contains an N-terminal ER luminal domain responsible for stress sensing and C-terminal kinase and endoribonuclease domain in the cytosol involved in splicing of XBP1 mRNA and in regulated IRE1-dependent decay (RIDD) (Figures 1 and 2). The luminal domain of the mammalian IRE1α crystallizes as a dimer with an overall architecture similar to the yeast protein (Zhou et al. 2006). A monomer of the luminal domain of IRE1α is composed of unique protein fold of a triangular-shaped β-sheet clusters, which provide a dimerization interface stabilized by hydrogen bonds and hydrophobic interactions (Zhou et al. 2006). Dimerization of the IRE1α luminal domain initiates autophosphorylation of the IRE1α cytosolic domain leading to activation of RNase activity (Zhou et al. 2006; Li et al. 2010). Moreover, dimerization of IRE1α creates a shared central groove that resembles a major histocompatibility complex-like fold allowing for peptide binding. This suggests that IRE1α can interact with peptides primarily composed of basic and hydrophobic residues that mimic misfolded proteins in the ER (Zhou et al. 2006; Gardner and Walter 2011). Mutation of amino acid residues within the groove prevents IRE1α interaction with peptides *in vitro* (Gardner and Walter 2011) and leads to impaired IRE1α signaling (Credle et al. 2005; Gardner and Walter 2011). Crystal structure of the cytoplasmic domains of IRE1α in the face-to-face (kinase active site points toward the active site of the opposite molecule) or back-to-back orientations provide important information for a mechanistic understanding of the function of IRE1α (Lee et al. 2008; Ali et al. 2011; Adams et al. 2019). These different orientations of the cytoplasmic domain may represent dynamic interactions between kinase and RNase activities of IRE1α to support its oligomerization and stress-induced signaling (Tirasophon et al. 2000; Korennykh et al. 2009; Itzhak et al. 2014).

Activation of RNase function of IRE1α requires dimerization-dependent intermolecular autophosphorylation (Tirasophon et al. 2000; Itzhak et al. 2014; Prischi et al. 2014). Mutations of IRE1α phosphorylation site reduce RNase splicing activity towards XBP1 mRNA (Prischi et al. 2014). Five amino acid residues within the RNase domain (D847, K907, G923, D927, and Y932) have been identified as essential for RNase activity but not kinase activity, and these mutations prevent activation of IRE1α (Tirasophon et al. 2000). These observations established an intrinsic mechanistic requirement for activation of IRE1α through the oligomerization of its kinase and RNase domains (Korennykh et al. 2009; Itzhak et al. 2014).

## The many functions of IRE1α

In response to ER stress, the luminal domain of IRE1α dimerizes/oligomerizes, and initiates trans-autophosphorylation of its cytosolic domain inducing a conformational change that leads to activation of IRE1α RNase activity located in the cytoplasmic domain (Liu et al. 2000; Zhou et al. 2006). RNase activity of IRE1α catalyzes the excision of 26 nucleotides within the mRNA encoding XBP1 transcription factor. This unconventional splicing event causes a frameshift resulting in a generation of a longer, stable, and activate transcription factor known as spliced XBP1 (XBP1s) (Yoshida et al. 2001; Calfon et al. 2002). XBP1s binds to a specific promoter element, known as the ER stress element and unfolded protein response element, and turns on expression of genes encoding proteins that modulate protein folding and, secretion, ERAD, protein translocation into the ER and lipid synthesis (Yoshida et al. 2001; Yamamoto et al. 2004). In addition, IRE1α can cleave multiple mRNA targets with consensus sequences and secondary structures that are similar to the XBP1 mRNA, via RIDD (Maurel et al. 2014). RIDD degrades RNAs, including mRNA encoding ER and cytosolic localized proteins, ribosomal RNA, and microRNAs, involved in many cellular functions such as energy metabolism, inflammation, and apoptosis (Maurel et al. 2014). Activation of RIDD preserves ER homeostasis or induces cell death but how IRE1α switches between cytoprotective to cytotoxic RIDD is not known (Lerner et al. 2012; Upton et al. 2012; Maurel et al. 2014). Among the three UPR signaling branches, IRE1α is the major trigger in ER stress-induced apoptosis, whereas PERK and ATF6 are dispensable in activation of apoptosis during prolonged ER stress (Upton et al. 2012). Sulfonation of IRE1α inhibits its signaling and activates p38/Nrf2 antioxidant responses under oxidative stress conditions (Hourihan et al. 2016).

IRE1α interacts with ER-associated inositol-1,4,5-trisphosphate receptor/Ca^2+^ channel (IP_3_R) and affects IP_3_R intracellular distribution and channel activity involved in the formation of functional ER-mitochondria contacts to transport of Ca^2+^ from the ER to the mitochondria (Agellon and Michalak 2019; Carreras-Sureda et al. 2019). Recently, two pools of IRE1α were identified in skeletal muscle and cardiomyocytes; one associated with junctional sarcoplasmic reticulum (SR) responsible for regulation of muscle excitation-contraction coupling and another in the ER-like perinuclear localized membrane system (Wang et al. 2019). |Junctional SR is enriched with the ryanodine receptor/Ca^2+^ channel (RyR) and calsequestrin, a Ca^2+^ binding muscle-specific protein (Wang and Michalak 2020). The RyR, at the junctional SR, is localized to membrane contacts enriched in L-type Ca^2+^ channel of the T-system, an invagination of the plasma membrane (Barone et al. 2015). Both RyR and L-type Ca^2+^ channel are critical for the regulation of Ca^2+^ released from the SR to trigger muscle contraction (Barone et al. 2015). As IRE1α is localized near both Ca^2+^ channels in muscle cells (Wang Q et al. 2019), it is tempting to speculate that IRE1α influences Ca^2+^ channel(s) function and, consequently, excitation-contraction coupling of muscle cells (Agellon and Michalak 2019). Interestingly, calsequestrin binds to IRE1α at the junctional SR preventing its oligomerization and splicing of the XBP1 mRNA (Wang et al. 2019) suggesting that IRE1α at the junctional SR represents different functions of the stress sensor. A role of IRE1α in the regulation of cellular Ca^2+^ signaling remains to be established.

## IRE1α interactome in the lumen of the ER

In the lumen of the ER, there are a number of multifunctional residents and integral membrane proteins that support many of the ER cellular functions including protein synthesis and post-translational modification, Ca^2+^ buffering/binding and signaling, the synthesis of lipids and steroids, regulation of gene expression, and energy metabolism (Benyair et al. 2011; Braakman and Bulleid 2011; Stutzmann and Mattson 2011). These proteins have access to the N-terminal luminal domain of IRE1α and some of them interact with IRE1α to influence its ability to detect or respond to ER stress (Table 1).

**Table 1.** IRE1α interacting proteins in the lumen of the ER/SR. In the lumen of the ER IRE1α forms functional complexes with proteins involved in ER Ca^2+^ signaling, protein syntheses, folding, and post-translational modification.

Protein	Function of interactors	Site of interaction with IRE1α	Impact on IRE1α function
BiP/GRP78 (Bertolotti et al. 2000; Kimata et al. 2007; Kimata et al. 2004; Okamura et al. 2000)	Immunoglobulin binding protein	A loop region proximal to the membrane	Binding to IRE1α under unstressed condition Key component of IRE1α ER stress sensing
Carboxypeptidase Y mutant (G255R) or ΔEspP-FAM (Credle et al. 2005; Gardner and Walter 2011)	Overexpressed misfolded proteins in the ER	Peptide binding groove, center of core IRE1α luminal domain	Activates IRE1α by increasing its oligomerization
PDIA6 (Eletto et al. 2014; Groenendyk et al. 2014)	Protein disulfide isomerase A6	Cys^109^, Cys^148^, and Cys^332^ in IRE1α ER luminal domain	Increases IRE1α phosphorylation and XBP1 splicing Forms a dynamic feedback loop with ER Ca^2+^ and miR-322 for IRE1α regulation
COX-2 (Groenendyk et al. 2018)	Cyclooxygenases-2	Not identified	Cyclosporine-dependent activation of XBP1 splicing
HSP47 (Sepulveda et al. 2018)	Heat shock protein 47, collagen chaperone	Not identified	Displaces BiP to activate IRE1α by promoting oligomerization and XBP1 splicing
Casq1 and Casq2 (Wang et al. 2019)	Skeletal muscle (Casq1) and cardiac (Casq2) Ca^2+^ binding protein in muscle SR	Not identified	Attenuates activation of IRE1α by preventing dimerization of IRE1α luminal domains
PRKCSH (Shin et al. 2019)	Protein kinase C substrate 80K-H and subunit of glucosidase II beta	Not identified	Enhances ER stress-mediated autophosphorylation and oligomerization of IRE1α Contributes to tumor resistance to ER stress
Sigma-1 receptor (Mori et al. 2013)	Resident protein in the ER-mitochondria contact site	Not identified	Interacts with IRE1α monomers Stabilizes IRE1α at mitochondria-ER-associated membrane (MAM) under ER stress
Cab45S (Chen et al. 2014)	45-kDa Ca^2+^-binding protein	Interacts with BiP	Stabilizes BiP interaction with IRE1α to inhibit ER stress-induced IRE1α activation and apoptosis
Sec61 (Plumb et al. 2015; Sundaram et al. 2017)	Component of the translocon	Region encompassing amino acid residues 434–443	Forms a hetero-oligomeric complex with IRE1α upon ER stress

### BIP

BiP, one of the most abundant ER-resident chaperones, was the first identified modulator of the IRE1α luminal domain (Bertolotti et al. 2000; Okamura et al. 2000). BiP interacts with ER luminal domain of IRE1α and prevents its dimerization and UPR signaling (Table 1). BiP also binds to the luminal domain of PERK and ATF6 under resting conditions and dissociates from PERK and ATF6 under ER stress (Bertolotti et al. 2000; Shen et al. 2002). These observations indicate that BiP is a common negative regulator of UPR by binding to the luminal regions of ER stress sensors (IRE1α, PERK, and ATF6) to maintain them in an inactive state.

Dissociation of BiP from IRE1α triggers activation of IRE1α to mediate UPR responses (Bertolotti et al. 2000; Okamura et al. 2000; Kimata et al. 2004). BiP dissociation from IRE1α may be mediated by interaction between BiP and misfolded proteins to sequester BiP away from IRE1α (Kopp et al. 2018; Adams et al. 2019). Alternative mechanisms have been put forward for IRE1α activation indicating that BiP dissociation may not be the sole criterion needed for activation of the IRE1α (Kimata et al. 2007; Oikawa et al. 2007; Pincus et al. 2010). For example, IRE1α may also be regulated by direct binding of unfolded protein (Gardner and Walter 2011; Amin-Wetzel et al. 2019), change in membrane lipid composition (Promlek et al. 2011), AMPylation of BiP [affected by ER Ca^2+^ (Veyron et al. 2019)], cooperation between BiP and ERdj4 (Amin-Wetzel et al. 2019), or yet unidentified factor(s).

### PDIA6

PDIA6, an ER-resident oxidoreductase, was identified as a regulator of IRE1α activity in response to depletion of the ER Ca^2+^ store (Eletto et al. 2014; Groenendyk et al. 2014). PDIA6 interacts with the luminal domain of IRE1α in a cysteine-dependent manner to enhance IRE1α activity (Table 1). Interestingly, PDIA6 does not substantially affect the activity of the PERK pathway that mediates responses to ER stress, suggesting that each arm of the UPR may be responsive to different components of the ER lumen. Importantly, ER store Ca^2+^ depletion and activation of store-operated Ca^2+^ entry reduces the abundance of the microRNA miR-322, which regulates PDIA6 mRNA stability and consequently IRE1α activity (Groenendyk et al. 2014). This is the first documented case for ER luminal Ca^2+^ together with PDIA6, IRE1α, and miR-322 functioning in a dynamic feedback loop regulating the UPR (Groenendyk et al. 2014).

### HSP47

HSP47 is an ER-resident foldase that belongs to the family of heat shock proteins and functions as a specific carrier for different types of collagens. It assists the transport of triple-helix procollagen from ER lumen to the cis-Golgi (Nagata 1996; Nagata Kazuhiro and Hosokawa 1996). Upon ER stress, HSP47 associates with the ER luminal domain of IRE1α reduces binding of BiP to IRE1α, promotes IRE1α dimerization/oligomerization and activates IRE1α-mediated UPR (Sepulveda et al. 2018). Importantly, HSP47 enhances the UPR upon ER stress specifically via the IRE1α signaling branch. Interestingly, overexpression or knockdown of HSP47 does not alter PERK and ATF6-mediated UPR signaling indicating HSP47 specificity for IRE1α (Sepulveda et al. 2018). In the heart transient activation of IRE1α results in severe fibrosis (Groenendyk et al. 2016). It is likely that HSP47-dependent activation of IRE1α plays a role in pathogenesis of cardiac fibrosis (Groenendyk et al. 2016).

### COX-2

Cyclosporine is an inhibitor of a Ca^2+^-dependent phosphatase, calcineurin, and it is widely used as an immunosuppressant drug (Azzi et al. 2013). Cyclosporine binds to cyclooxygenase-2 (COX-2) and chronic exposure to cyclosporine causes nephrotoxicity and organ damage. COX-2, an inducible cyclooxygenase that drives inflammation, interacts with the ER luminal domain of IRE1α and enhances its XBP1 splicing (Groenendyk et al. 2018). Cyclosporine triggers activation of IRE1α through binding to COX-2, which forms a complex with IRE1α (Groenendyk, Paskevicius, Urra, et al. 2018). Cyclosporine associates to COX-2 resulting in enhanced COX-2 enzymatic activity that is required for IRE1α activation. This offers a novel mechanism for cyclosporine-induced IRE1α signaling (Groenendyk, Paskevicius, Urra, et al. 2018).

### Calsequestrin

Calsequestrin (skeletal muscle and cardiac calsequestrin PDIAB1 and PDIB2, respectively), another PDI-like family of protein, is a muscle-specific Ca^2+^ binding and storage protein in the SR (Costello et al. 1986; Wang S et al. 1998; Eisner et al. 2017). Recently, we discovered that both skeletal muscle and cardiac calsequestrin bind to the IRE1α luminal domain in the SR where it modulates IRE1α activity (Wang Q et al. 2019). Association between calsequestrin and IRE1α prevents IRE1α dimerization/oligomerization, an initiation step in IRE1α activation, making calsequestrin a muscle-specific modulator of IRE1α (Wang Q et al. 2019).

Taken together, these findings reveal crucial role of the ER/SR luminal proteins in providing multiple level of regulation of stress sensing and stress responses.

## IRE1α interacting partners in the cytosol

Most studies on regulation of the IRE1α signaling pathway have focussed on the cytoplasmic regulators of IRE1α activity (Table 2). While the ER luminal domain of IRE1α is important in stress sensing, IRE1α activation is tightly controlled by a number of proteins interacting with its cytoplasmic domain including phosphatases, kinases, apoptosis-related proteins, and the cytoskeleton (Table 2) (Hetz 2012; Chen and Brandizzi 2013; Groenendyk et al. 2013; Riaz et al. 2020). IRE1α cytosolic domain interacting proteins enhance or inhibit IRE1α RNase activity, or act as a scaffold and recruit other proteins to activate apoptosis signaling (Table 2) (Hetz and Glimcher 2009; Chen and Brandizzi 2013). For example, the cytosolic domain of oligomerized IRE1α binds to the adapter protein TNFR-associated factor 2 (TRAF2), triggering activation of the apoptosis signal-regulating kinase 1 (ASK1) and c-Jun-N-terminal kinase (JNK) pathway (Urano et al. 2000; Nishitoh et al. 2002). IRE1α-TRAF2 also promotes NF-κB in a TNFR1-dependent manner and is dependent on the autocrine production of TNFα. Phosphorylated JNK stimulates the cytochrome c-mediated apoptotic pathway by phosphorylating different members of the Bcl-2 family of proteins (Tournier et al. 2000; Lei and Davis 2003).

**Table 2.** Molecules interacting with IRE1α in the cytosol. IRE1α forms functional complexes with molecules involved in cellular metabolism, apoptosis and signaling.

Protein name	Interactors	Site of interaction with IRE1α cytosolic domain	Impact on IRE1α function
Fortilin (Pinkaew et al. 2017)	Translationally controlled tumor protein	Binds phosphorylated IRE1α at S^724^ and S^726^	Binds to phosphorylated IRE1α Inhibits IRE1α kinase and RNase activities Protects cells against ER stress-induced apoptosis
BAX/Bak (Hetz et al. 2006)	Proapoptotic BCL-2 family members	Not identified	Activates IRE1α signaling
BI-1 (Lisbona et al. 2009)	Apoptosis regulator Bax inhibitor 1	Not identified	Inhibits IRE1α activation Reduces binding of BAX to IRE1α
TRAF1 (Urano et al. 2000)	Tumor necrosis factor receptor	Not identified	Mediates IRE1α dependent activation of the stress-activated protein kinase/c-Jun N-terminal kinase (JNK)
TRAF2 (Castillo et al. 2011; Urano et al. 2000)	Tumor necrosis factor receptor-associated factor 2	Not identified	Facilitates recruitment of JNK to IRE1α to induce apoptotic signaling
JIK (Yoneda et al. 2001)	c-Jun N-terminal inhibitory kinase	Not identified	Modulates IRE1α and TRAF2 complex formation; Induces apoptotic signaling through JNK pathway and activation of caspase-12
UBD (Brozzi et al. 2016)	Protein ubiquitin D Ubiquitin-like modifier family member	Not identified	Modulate IRE1α dependent activation of JNK and cytokine-induced apoptosis
ASK1 (Nishitoh et al. 2002)	Apoptosis signal-regulating kinase 1	Not identified	Forms complex with IRE1α and TRAF2
Aip-1 (Luo et al. 2008)	Ask1 interacting protein 1	Not identified	Promotes oligomerization and activation of IRE1α signaling
RNF13 (Arshad et al. 2013)	RING finger protein 13	Not identified	Facilitates ER stress-induced apoptosis via activation of the IRE1α-TRAF2-JNK signaling pathway
NMI (Brozzi et al. 2014)	N-Myc interactor	Not identified	Associates with IRE1α in pancreatic beta cells Negatively regulates IRE1α-dependent activation of JNK and apoptosis
PARP16 (Jwa and Chang 2012)	Poly(ADP-ribose) polymerase, ER transmembrane protein	Not identified	Activate IRE1α kinase and RNase activities
HSP72 (Gupta et al. 2010)	Stress-inducible cytosolic chaperone	Not identified	Enhance IRE1α RNase activity and inhibits ER stress-induced apoptosis
PTP-1B (Gu et al. 2004)	Protein-tyrosine phosphatase 1B	Not identified	Required for ER stress-induced apoptosis
NMHCIIB (He et al. 2012)	Nonmuscle myosin heavy chain IIB, a subunit of nonmuscle myosin IIB	Not identified	Promotes IRE1α oligomerization
Filamin A (Urra et al. 2018)	Actin crosslinking factor	Not identified	Interacts with monomeric IRE1α and regulates cell migration independent of XBP1 splicing
Hsp90 (Marcu Monica et al. 2002)	Heat shock protein 90	Not identified	Stabilizes IRE1α protein by preventing the proteasomal degradation
JAB1 (Oono et al. 2004)	Jun activation domain-binding protein-1	Linker region of IRE1α cytoplasmic domain (amino acid residues 507-550)	Binds to IRE1α in the absence of stress but dissociate upon stress induction.
RACK1 (Qiu et al. 2010; Liu et al. 2016)	Scaffold protein receptor for activated C-kinase 1	Not identified	Interacts with IRE1α upon glucose stimulation Inhibits glucose-stimulated IRE1α activation Attenuate IRE1α-dependent increases in insulin production
Nck (Nguyên et al. 2004)	Non-catalytic region of tyrosine kinase adaptor protein	Not identified	Binds to IRE1α In T-cells activates MAPK pathway and cell survival
BIM and PUMA (Rodriguez et al. 2012)	Proapoptotic BH3-only proteins P53 upregulated modulator of apoptosis (PUMA)	Not identified	Cells deficient in BIM and PUMA shown reduced XBP1 splicing and RIDD
Dcr2 (Guo and Polymenis 2006)	Dose-dependent cell-cycle regulator 2	Not identified	Interacts with phosphorylated IRE1α
SYVN1 (Gao et al. 2008)	E3 ubiquitin ligase synoviolin Anti-apoptotic factor	Not identified	Promotes IRE1α degradation and ubiquitination Antagonizes ER stress-induced cell death
DDRGK1 (Liu et al. 2017)	DDRGK domain-containing protein 1	Kinase domain of IRE1α	Interacts with non-phosphorylated IRE1α Increases IRE1α protein stability
ABL kinase (Morita et al. 2017)	Tyrosine-protein kinase	Not identified	Enhances IRE1α RNase activity Promotes IRE1α apoptosis signaling pathway

## Summary points

- Structural studies revealed mechanistic requirements for IRE1α activation.
- IRE1α is found in membrane contact sites where it regulates organellar communication.
- ER luminal proteins responsible for ER Ca^2+^ signaling, protein synthesis, folding and modification interact with IRE1α to regulate its functions.
- In the cytosol IRE1α is regulated by molecules involved in cellular metabolism, apoptosis and signaling.
